# Treatment of Meniere’s Disease

**Published:** 1881-06

**Authors:** 


### TREATMENT OF MENIERE'S DISEASE.

Dr. M. E. Meniere (*La France Med.*, 1881, p. 329) has recently published a brochure on the affection described in 1861 by his father and which has since been called by his name. The disease, as is known, is characterized by three principal symptoms: 1, noises or whistling in the ear, which precede the attacks; 2, vertigo, accompanied by nausea and vomiting; 3, final deafness, which is usually incurable. Without going into a full description of the disease, Dr. Meniere, the younger, chiefly writes of the treatment, following the plan proposed by Prof. Charcot. The patient takes the following at meal times: ℞ Quiniæ sulphat., Ext. cinchonæ, āā gr. jss. Beginning with this pill to the amount of about five grains of sulphate of quinine daily, the daily amount is increased to twelve or fifteen grains. Then no medicine is taken for from one to three weeks, and then the quinine is commenced again in the dose of six grains a day and continued for a month. The effect of the quinine is to diminish the vertigo and finally to cause it to disappear entirely, modifying also to some degree the deafness. M. Meniere does not pretend to be able to formulate a cure for a disease in which all the resources of therapeutics have thus far proved vain, but quinine has at least the advantage of reducing some of the more severe symptoms.

